# The legal personhood of human brain organoids

**DOI:** 10.1093/jlb/lsad007

**Published:** 2023-04-03

**Authors:** Masanori Kataoka, Tsung-Ling Lee, Tsutomu Sawai

**Affiliations:** Graduate School of Humanities and Social Sciences, Hiroshima University, Hiroshima, Japan; Graduate Institute of Health and Biotechnology Law, Taipei Medical University, Taipei, Taiwan; Graduate School of Humanities and Social Sciences, Hiroshima University, Hiroshima, Japan; Institute for the Advanced Study of Human Biology, Kyoto University, Kyoto, Japan

**Keywords:** brain organoids, legal status, legal personhood

## Abstract

Research using three-dimensional neural tissues derived from human pluripotent stem cells—known as ‘human brain organoids’—has progressed rapidly in recent years. Although related ethical issues have been intensively discussed, legal issues have only been sparsely examined compared with the related ethical issues. In this paper, we explore a fundamental issue concerning the legal status of human brain organoids: whether they can be considered legal persons. We clearly distinguish between two types of legal personhood: ‘natural person’ as a human legal person and ‘juridical person’ as a nonhuman legal person. By examining natural and juridical personhood separately, we point out the bias and confusion in the remarks on the legal personhood of human brain organoids and provide a more comprehensive picture of the problem.

## I. INTRODUCTION

Research using three-dimensional neural tissues derived from human pluripotent stem cells—known as ‘human brain organoids’—has progressed rapidly in recent years. As such research has been reported, national and international bodies have raised various ethical issues, discussing whether and how regulation is required.^1^ However, although the ethical issues have attracted much attention and been intensively discussed, the accompanying legal issues have only been sparsely examined.

There are various legal issues relating to human brain organoid research, which can be grouped into three main types: first, how the source materials ought to be obtained; second, the legal status of human brain organoids; and third, questions relating to the various possible uses from academic to commercial.^2^ These three issues, however, are not entirely independent of one another since the first and third depend heavily on the legal status of human brain organoids. To clarify the legal status of human brain organoids will illuminate issues such as what information should be informed to the cell donor, to what extent the donor’s consent justifies the research, and what uses are acceptable. Of course, it also defines what accountability researchers and institutions have for their research. In addition, although the questions of material acquisition and uses are issues for all types of organoids, legal status is more specific to brain organoids. Indeed, at least at first, it may seem that human brain organoids are more than just tissues. These are structural and functional recapitulations of the human brain, albeit imperfect in their present state. Therefore, they may have important functions of the human brain. For example, if they have various vital functions, they can be considered as an organism rather than just a tissue. Alternatively, if they experience pain, they may be worthy of protection. Examining these naïve intuitions more closely and considering the legal status of human brain organoids is important for the legal evaluation of human brain organoid research.

In this paper, we examine one of the most fundamental issues concerning the legal status of human brain organoids: whether they can be considered legal persons. Since the term ‘legal person’ is often used ambiguously, along with several related terms, we provide a preliminary explanation of our terminology here, not only to make the paper more readable (and understandable), but also with a view to clarifying some of the confusions and incompleteness of existing discussions. We use the term ‘legal person’ as a generic term referring to any entity that holds legal rights and obligations. In this sense, a legal person can be a human or nonhuman entity. The former, a human legal person, is called a ‘natural person,’ whereas the latter, a nonhuman legal person, is called a ‘juridical person’—common examples include corporations, governmental agencies, and nongovernmental organizations. In addition, we distinguish legal personhood from moral personhood (moral agency): although the two are not necessarily unrelated, we do not make any normative claims about legal personhood from moral perspectives. Instead, our discussions relate solely to current legal practices (*de lege lata*) or ongoing legal debates.

Although several authors have discussed the problem of legal personhood in relation to human brain organoids, they have mainly focused on whether human brain organoids can be natural persons.^3^ However, even if human brain organoids cannot be natural persons, they can still be legal persons in another sense since they can be juridical persons. The conditions for an entity to be a natural person are quite different from those for an entity to be a juridical person. Therefore, the question as to whether human brain organoids can be classified as legal persons comprises two sub-questions. First, can human brain organoids be natural persons? Second, can human brain organoids be juridical persons? We examine both questions below.

In Section II, we examine the possibility of human brain organoids being natural persons. Such a possibility has previously been discounted based on the facts that human brain organoids cannot be integrated into the body as a whole and that they are not legally ‘born’ in the first place. We believe that these criticisms are valid in relation to current human brain organoids, but we also argue that this possibility should soon be revisited, for several reasons. First, research into linking human brain organoids with living or non-living bodies is expected to advance rapidly in the future. Second, the concept of legal birth is flexible and, currently, controversial. Third, these issues need to be discussed sooner rather than later, in light of the potential use of human brain organoids for human reproductive cloning.

In Section III, we then consider the possibility of human brain organoids being nonhuman juridical persons. We begin by pointing out that some major theories of juridical personhood do not necessarily rule out the juridical personhood of human brain organoids. We next review recent trends relating to the juridical personhood of animals, rivers, and artificial intelligence systems, arguing that the rationales for granting juridical personhood to such entities also apply to human brain organoids. Finally, based on the legal distinction between natural and juridical personhood, we argue against the claim that granting legal personhood to human brain organoids would reduce human dignity.

## II. HUMAN BRAIN ORGANOIDS AS NATURAL PERSONS

### II.A. The Argument from the Brain Death Criterion

According to a recent interview study, some citizens feel that there is a continuity between the creation of human brain organoids and the potential for human cloning.^4^ This is quite natural, given the nature of human brain organoids: they are indisputably of human origin, and the brain is generally considered to be the most critical element of a human being.

However, to our knowledge, there is only one *argument* for the claim that human brain organoids can be classified as human beings legally or as natural persons. The argument proposed by Andrea Lavazza and Federico Gustavo Pizzetti is as follows.^5^ In many countries, the legal end of a natural person’s life is defined either by the irreversible cessation of the heart’s beating or by brain death—when one’s brain irreversibly stops all neural activities, one is legally dead at that point. Given this criterion, if a human brain organoid can stimulate the relevant neural activities, then surely it is alive as a natural person? Let us call this argument the ‘argument from the brain death criterion.’ According to this argument, the scenario of human brain organoids becoming natural persons is increasingly imminent because researchers have already identified human brain organoids as stimulating neural activities comparable to those of the human brain.^6^

However, this argument can be criticized for two reasons.^7^ The first objection concerns the type of neural activity that the brain death criterion presupposes. What is at issue here is not the termination of any neural activities, but rather the termination of the neural activities that integrate the human body as a whole. This acknowledges an important element of what a natural person is: a natural person is an integrated organism, and the brain is essential to this organism since it performs the integrating. Thus, by analogy with the brain death criterion, for a human brain organoid to be considered a natural person, it is not enough for the organoid to simply exhibit neural activities—instead, the human brain organoid must have the capacity to integrate the body as a whole. Current (immature) human brain organoids do not possess such a capacity, and it is believed that they will only acquire such a capacity, if it proves to be possible, in the distant future.

Second, for an entity to be a natural person, it must first be ‘born’ in the legal sense. Legal birth is typically defined by the partial or complete expulsion of a fetus from a womb. Human brain organoids do not meet this requirement, for two obvious reasons. To begin with, human brain organoids are not equivalent to a fetus or even to an embryo, since, unlike them, human brain organoids do not have the potential to develop into a whole human organism.^8^ Furthermore, and more obviously, the brain organoids are not expelled from a womb, as they are cultivated *in vitro*. This last point is fundamental: no matter how mature and cognitively advanced human brain organoids may become in the future, they simply cannot be classified as natural persons under current laws because they are not born from a womb.

### II.B. Further Considerations

These criticisms against the argument from the brain death criterion are entirely plausible in relation to current human brain organoids. However, we believe that the plausibility of these criticisms should be called into question sooner rather than later. There are several reasons: the current directions of human brain organoid research; the flexible understanding as to what constitutes the ‘body’ of a natural person in various current legal practices; the ongoing controversies relating to the legal concept of birth as expulsion from a womb; and the potential for human reproductive cloning. We explore each of these reasons below.

#### 1. Directions of Research

First, research into linking human brain organoids with human ‘bodies’ is likely to proceed rapidly in the future. Researchers have frequently pointed out that one of the major limitations of current human brain organoids is their lack of sensory inputs and motor outputs. This is an obstacle to the use of human brain organoids as models for the neurodevelopment of the human brain. Therefore, linking living or non-living bodies to human brain organoids is a significant goal of future research.^9^

In a recent study, human brain organoids were functionally integrated with optic vesicles and made to respond to light stimuli.^10^ Another study fused human cerebral and spinal cord organoids with human muscle spheroids (cell aggregates).^11^ Yet another study connected seven types of organoids, including brains, into a ‘human-on-a-chip’ that mimics actual human physiology more closely.^12^ These studies are still in their early stages, but it is safe to say that future human brain organoid research will involve further attempts to link human brain organoids to human bodies. Indeed, the day when human brain organoids will have the capacity to integrate the body in one sense or another may be on the horizon.

#### 2. What Kind of Body?

Nonetheless, there are likely to be so many challenges in functionally connecting a human brain organoid to the equivalent of a natural person’s body at typical birth i.e. a fetus at 40 weeks of age. Due to this difficulty, we suspect, it is often claimed that human brain organoids will only acquire the capacity to integrate the whole body in the very distant future. However, this brings us to our second point: what exactly should the ‘whole body’ or ‘whole organism’ mean in this context? If an entity could qualify as a natural person simply by having the capacity to integrate a relatively simple body, then human brain organoids may meet this condition in the near future.

Even today, infants born before the 40 weeks of gestation can be legally considered natural persons. For example, according to the Born Alive Rule, a common law principle in England and related countries, infants can be considered natural persons as long as they are born alive. This means that the lower limit of physical maturity for a natural person is ultimately determined by viability—that is, the ability of the fetus to survive after delivery. The fetus is generally thought to acquire this ability at around 22–24 weeks after conception. Certainly, the body of a 22-week-old infant is already fairly complex, but even this standard is not always indisputable. In fact, the United States has explicitly rejected this idea in the Born-Alive Infants Protection Act of 2002.^13^ According to this Act, regardless of the likelihood of the infant’s lasting survival outside the womb, a born infant is a natural person ‘at any stage of development,’ as long as that infant displays signs of breath, a heartbeat, the pulsation of the umbilical cord, or voluntary muscle movements.^14^

Thus, at least for the natural personhood of infants, the required body complexity is flexible, and there are moves to loosen it. If this can be applied beyond infants to human brain organoids, then human brain organoids can become natural persons simply by having the capacity to integrate a relatively simple ‘whole body.’ Especially under standards such as the Born-Alive Infants Protection Act of 2002, which emphasizes only very basic vital functions, human brain organoids may be considered natural persons in the near future. This is because basic vital functions, such as breathing and heartbeat, are related to the brainstem, and brainstem organoids have already been created.^15^

#### 3. Is There a Need to Be Born?

Previous discussions about the maturity of the body of natural persons have assumed that the infant is born. In the legal sense, ‘birth’ generally means the expulsion of a fetus from a womb. Given that human brain organoids are not born in this sense, however, they cannot be considered natural persons, no matter how capable they are of integrating whole bodies.^16^

Once again, though, this traditional conception of birth is by no means unquestionable, and it has been most contested in relation to the issue of abortion: we do not wish to debate the controversies surrounding this complex issue here, but rather to emphasize that the traditional conception of birth is already being questioned from another perceptive.

Recently, fetal surgery has become possible. It is a surgical procedure whereby a previable fetus is removed from the womb, medical interventions are performed (such as tumor removal), and then the fetus is returned back to the womb. This development has made the legal conception of birth as the expulsion of a fetus from a womb very confusing.^17^ Under current laws, a fetus that is removed from a womb for treatment, even only temporarily, must be considered as being legally born, because it has been expulsed from a womb. In the above procedure, then, a newborn—and not a fetus—is returned to the womb, meaning that there is now a natural person inside the mother’s body. At this time, since it is a newborn in the mother’s body, the mother becomes their legal guardian and thus must act for their best interest. But this conflicts overtly with the pregnant women’s right of self-determination, which is usually protected clearly by other laws or precedents. This confusion forces us to reconsider the traditional understanding that the expulsion of a fetus from a womb marks the beginning of a natural person. What is more, related problems will arise in the future with the development of artificial womb technology.^18^

Therefore, the conditions for natural personhood are relatively flexible and under ongoing debates. We do not intend to commit ourselves to any particular positions in relation to these various controversies over abortion, fetal surgery, or artificial wombs; rather, we wish to emphasize that such controversies are relevant to human brain organoids and accompanying questions of natural personhood. If developments in sciences and technologies ultimately mean that conditions such as viability and birth—the expulsion of a fetus from a womb—are no longer necessary for an entity to be considered a natural person, this will weaken the ground for arguing that a human brain organoid that can integrate a relatively immature body is not a natural person. Indeed, recent abortion trends in the United States have called for less strict conditions for natural personhood. As such, it is important to seriously examine the possibility that human brain organoids could soon be considered natural persons.

#### 4. Is This Human Reproductive Cloning?

Finally, it is important to seriously consider the likelihood of human brain organoids having the potential to become natural persons because this issue is closely related to the question of human reproductive cloning. As such, it cannot be misjudged.

In many countries, human reproductive cloning is prohibited as a violation of human dignity—one of the most fundamental values of human beings. Currently, however, the establishment of human brain organoids is not considered human reproductive cloning.^19^ On the one hand, human reproductive cloning is a technique that produces *an individual human being* genetically identical to another human being. On the other hand, human brain organoids have the same genetic information as their cell donors, unless the genes in the human brain organoids are artificially edited, but they are only *portions of tissue*, rather than full human beings. Therefore, human brain organoids may be legally created in many countries that outlaw human reproductive cloning. We believe that such an understanding of human brain organoids is appropriate now.

However, the situation may be different in the future for more mature brain organoids. The stronger the brain organoid’s capacity to integrate the body, the more challenging it becomes to consider it as just a portion of tissue. Especially, how should we comprehend the following scenario, which is not so implausible, that a human brain organoid is linked and sufficiently integrated with various tissues or organs that have been derived from the same stem cells from which the human brain organoid was derived? If this were to be achieved, the whole complex would share its genetic information with a single donor. It is not clear how, exactly, this would differ from human reproductive cloning.

To summarize this section, although human brain organoids do not constitute natural persons at present, the likelihood of their potential to become natural persons in the near future requires more thorough consideration in advance of that reality occurring. Research on linking human brain organoids with bodies is expected to advance rapidly in the coming years, whereas the conditions of natural personhood, especially viability and birth are becoming increasingly flexible and contentious. Furthermore, this issue involves the most fundamental value of human dignity, making the issue so important that we cannot get the decision wrong.

## III. JURIDICAL PERSONHOOD

### III.A. Theories of Nonhuman Juridical Personhood

To the best of our knowledge, previous discussions about the legal personhood of human brain organoids have focused almost exclusively on natural personhood. However, as is obvious when one considers the firm, an entity can still be a juridical person without being a natural person. Thus, discussions about the legal personhood of human brain organoids are incomplete unless they also examine whether such an organoid can be considered a juridical person. Since the answer depends upon understanding what a juridical person is, it will be useful to touch upon some of the major theories that explain how a corporation, as the least problematic example, can be considered a juridical person before examining their applicability to the human brain organoids.^20^

According to the ‘aggregate theory,’ a corporation is nothing but an aggregation of stakeholders, and its juridical personhood is merely a symbol of the various legal relationships between these stakeholders.^21^ Since brain organoids are not constituted of stakeholders (as people), it would be difficult for a brain organoid to be considered a juridical person under this theory.

By contrast, the ‘real or natural entity theory’ understands a corporation as an entity that exists independently of its stakeholders and deserves juridical personhood due to its own characteristics. Current immature brain organoids do not possess any psychological properties, so it would be somewhat difficult to recognize their juridical personhood in accordance with their own characteristics. However, future brain organoids may come to possess various psychological properties, such as pleasure or pain, and if they become subjects of welfare, this could provide a basis for their juridical personhood. This point will be further examined below.

Finally, the ‘artificial person theory’ claims that a juridical person is a purely fictional entity that is created by laws for specific juridical purposes. This can circumvent ontological questions about the entities that are to be juridical persons. Nonetheless, there must be some practical legal purposes in granting juridical personhood. This theory would therefore appear to be most applicable to human brain organoids once a critical question has been answered: what would be the legal purposes of granting human brain organoids juridical personhood?

Some major theories of juridical personhood do not necessarily preclude the possibility of human brain organoids having juridical personhood. It should also be pointed out that in practice, different theories are employed in different cases, even within a single state. Even so, can the juridical personhood of human brain organoids be recognized for positive reasons? To answer this question, let us take some further examples of nonhuman juridical persons and examine the rationales for recognizing their juridical personhood, which could provide some suggestions for considering the juridical personhood of human brain organoids.

### III.B. Positive Reasons for Recognizing Juridical Personhood

#### 1. Animals

One frequent concern about human brain organoids is that they will be able to feel pleasure or pain in the future—that is, they will be sentient. Since sentience is often considered the minimum requirement for an entity to be a subject of welfare, questions have accordingly been raised about how the welfare of such potentially sentient human brain organoids should be protected. So far, this point has mainly been discussed as an ethical issue.^22^ And when it has been addressed in relation to laws, the focus is on possible legal protections for the welfare of sentient human brain organoids, often by analogy with existing animal welfare laws.^23^

In general, legal protection of the welfare of sentient animals does not require that they be granted juridical personhood. However, a transition from the former to the latter has been observed in several cases. For example, the United States District Court for the Southern District of Ohio recently recognized the juridical personhood of a group of hippopotamuses—for the first time in the United States.^24^ These animals, which live along the Magdalena River, were recognized as plaintiffs in a lawsuit against the Colombian government, which planned to either kill or sterilize them. Such recognition has been considered a natural extension of the rights that have already been granted to animals by means of animal cruelty laws and others.

This decision, then, can have important implications for the juridical personhood of human brain organoids. It has already been pointed out that two of the rationales for animal welfare laws are, at least theoretically, equally applicable to the potentially sentient human brain organoids of the future: the protection of their welfare and the strength of the public feeling about suffering of organoids.^25^ If human brain organoids were to be protected in similar ways as animals are protected by animal welfare laws, this could be a first step toward recognizing their juridical personhood.

#### 2. Rivers

Rivers constitute another important set of precedents for us. In recent years, rivers have become juridical persons in several countries, with the most famous example being the Whanganui River in New Zealand, whose juridical personhood was granted by the state in 2017.^26^ This recognition of juridical personhood was based on the worldview and values of the Maori, who understand that the Whanganui River to be a living entity, which is not something that people can own and that people have specific responsibilities toward it. This case highlights how social beliefs and values can play an important role in nonhuman entities being recognized juridical persons, rather than mere properties.^27^

The last point makes us aware of an often-missed point in the normative discussions about human brain organoids. So far, there has been much discussion as to whether human brain organoids can feel pleasure or pain, and the potential moral implications. By contrast, the question of how *people* think about and evaluate human brain organoids has hardly been examined. However, such public attitudes could be a key to human brain organoids being granted juridical personhood. Even though brain organoids are not natural persons and do not possess consciousness, people may be particularly concerned about such entities originating from humans and mimicking the human brain, leading them to regard human brain organoids as individual and independent entities deserve to be treated responsibly. Indeed, in the two interview studies, people tended to regard human brain organoids as morally distinct entities.^28^ Consequently, it would not be impossible for human brain organoids to be granted juridical personhood as a legal reflection of such public attitudes.^29^ In any event, there is no doubt that further research on public attitudes toward human brain organoids will be necessary to fully examine juridical personhood.^30^

It is also important to note that as a juridical person, the Whanganui River has appointed guardians who act for the river’s interests. The same might occur if human brain organoids were given juridical personhood. The guardians would then have the role of ensuring that the research is conducted responsibly and ethically.

#### 3. Artificial Intelligence Systems

Finally, it is also useful to consider recent active debates about the juridical personhood of artificial intelligence (AI) systems. Comparisons between AI systems and human brain organoids are reasonable. A recent study incorporates neurons derived from human induced pluripotent stem cells into silicon chips, and the resulting system can learn to play a simple video game.^31^ In the future, such biocomputing technology will be able to develop and utilize human brain organoids in further ways. However, although the ethical issues relating to such technology were quickly pointed out,^32^ the legal implications have yet to be examined. The same legal issues as those surrounding AI systems today will probably arise in relation to future organoid intelligence systems, including questions of juridical personhood.^33^

There are multiple fields in which the juridical personhood of AI systems will be at issue, with self-driving vehicles and other autonomous systems being the most well-known examples. The European Parliament once suggested that highly sophisticated autonomous systems could be considered juridical persons to account for any harms they bring about.^34^ A similar consideration could potentially apply to organoid intelligence systems, should they be able to make decisions that have significant impacts on human health, for instance. That said, it will likely be a long time before human brain organoids can conduct accurate calculations with the complexity required for self-driving cars, and it may not even be possible for them to do so.

What is more feasible will be to generate images or musical compositions by means of human brain organoids, raising questions of juridical personhood in relation to intellectual property rights. It is presently the case that many countries have made unfavorable rulings in terms of AI systems being able to hold copyrights or patents as juridical persons,^35^ and similar decisions may be made in the future with regard to the various creations of organoid intelligence systems. In any way, we wish to emphasize that such current debates about the juridical personhood of AI systems could potentially, as an unintended side effect, affect future debates about the juridical personhood of human brain organoids.

In summary, questions about the potential juridical personhood of human brain organoids have so far been overshadowed by a focus on natural personhood. However, natural personhood and juridical personhood are independent concepts that need to be considered separately. As we have pointed out, there can be positive reasons for granting juridical personhood to human brain organoids, and this question should continue to be explored in parallel with similar legal discussions in related areas.

### III.C. A Reduction of Human Dignity?

Finally, we address an argument that conflicts with our generally positive attitude toward the possible juridical personhood of human brain organoids. In a lengthy footnote to their paper, Lavazza and Pizzetti refer to recent debates concerning the legal personhood of AI systems, then consider whether similar considerations could apply to human brain organoids, just as we have done above.^36^ However, they proceed to argue against the possible legal personhood of human brain organoid, based on the fact that, unlike AI systems, human brain organoids are of human origin. The argument is as follows. Human brain organoids are human tissues that are detached from human beings as a whole. As such, if human brain organoids were to be granted legal personhood, this would mean that detached body parts and human beings as a whole would be considered legally ‘equivalent.’ This would ultimately amount to a reduction of human dignity because human dignity, they argue, should uniquely be given to individual human beings.

If successful, this objection would offer a strong reason against granting legal personhood to human brain organoids. However, it is not plausible. If granting legal personhood to individual parts of the human body were to result in a reduction of human dignity, then granting legal personhood to nonhuman entities, like companies, must indeed constitute a far more significant reduction of human dignity, because it considers human beings and nonhuman entities to be legally equivalent. However, no one would think that the legal personhood of companies would offend human dignity.

We think the objection from human dignity confuses juridical personhood with natural personhood, under the generic term ‘legal personhood.’ If laws were to recognize individual parts of the human body as natural persons, in the same way that they currently recognize individual human beings as natural persons, then yes, this would constitute a reduction of human dignity.^37^ However, granting *juridical* personhood to individual parts of the human body is compatible with granting *natural* personhood to the whole human being. Here the parts and the whole are not legally ‘equivalent.’

## IV. CONCLUSION

Research into human brain organoids is of great importance, since it will advance our understanding of the human brain and have a wide range of potential applications. Nonetheless, the fact that human brain organoids are created from human pluripotent stem cells, by harnessing the developmental processes of the human brain, continues to raise various ethical concerns, which many papers have discussed. Ethical considerations are necessary for research to be appropriately regulated, but legal considerations are also important. Indeed, many have addressed the need to establish appropriate ethical *and legal* frameworks for brain organoids research. So far, however, the legal issues have not received sufficient consideration relative to the ethical issues.

This paper has therefore focused on the legal status of human brain organoids, the most basic of these legal issues, and examined whether human brain organoids can be considered legal persons. Although some have discussed this issue, they have not drawn clear distinctions between the two types of legal personhood: natural personhood and juridical personhood. As a result, the earlier discussions are biased toward natural personhood implicitly, and can be conceptually confusing.

Based on the distinction between natural and juridical personhood, we have broached various issues relating to the legal personhood of human brain organoids. Current brain organoid technology is in many ways quite limited, and it has not yet reached a stage where human brain organoids could become natural or juridical persons.^38^ However, as we have emphasized, this issue will soon become urgent, once brain organoid technology has been further developed. In preparation for that time, it is essential to examine the accompanying questions thoroughly and in advance; we have taken the first step in that direction.

Although this paper provides an overall picture of the problem, it does not examine the individual issues in depth. Similarly, we have presented the arguments in a very general form, without discussing the specific laws of any country or region, relying on illustrative examples from a few countries. In the future, this problem will need to be more closely examined in relation to the specific legal systems of individual countries and regions.

Aside from the question of legal personhood, many legal issues surround current and future research into human brain organoids. The literature has already pointed out the legal issues relating to sample acquisition, ownership, commercialization and patenting, the protection of the welfare of human brain organoids, their transplantation into animals, neuroenhancement, and the potential global harmonization of legal standards, among other things.^39^ Since these issues are all closely related to the legal status of human brain organoids, it will be necessary to examine them more thoroughly in relation to the question of legal personhood and its various consequences.

## Footnotes

1 International Society for Stem Cell Research, *ISSCR Guidelines for Stem Cell Research and Clinical Translation*, https://www.isscr.org/guidelines (2021) (accessed March 6, 2023); National Academies of Sciences, Engineering, and Medicine, The Emerging Field of Human Neural Organoids, Transplants, and Chimeras: Science, Ethics, and Governance (2021).

2 Jochen Taupitz, *What Is, or Should Be, the Legal Status of Brain Organoids*?, in Brain Organoids in Research and Therapy: Fundamental Ethical and Legal Aspects 99–117 (Hans-Georg Dederer & David Hamburger eds, 2022).

3 Sorin Hostiuc, Mugurel Constantin Rusu, Ionuţ Negoi, Paula Perlea, Bogdan Dorobanţu & Eduard Drima, *The Moral Status of Cerebral Organoids*, 10 Regen. Ther*.* 118–22 (2019); Henry T. Greely, *Human Brain Surrogates Research: The Onrushing Ethical Dilemma*, 21 AM. J. Bioeth. 34–45 (2021); Andrea Lavazza & Federico Gustavo Pizzetti, *Human Cerebral Organoids as a New Legal and Ethical Challenge*, 7 J. Law Biosci. lsaa005 (2020); Taupitz *op*. *cit*., pp. 100–3.

4 Dolly R. Haselager, Sarah N. Boers, Karin R. Jongsma, Christiaan H. Vinkers, Marike L. Broekman & Annelien L. Bredenoord, *Breeding Brains? Patients’ and Laymen’s Perspectives on Cerebral Organoids*, 15 Regener. Med. 2351–60 (2020).

5 Lavazza & Pizzetti *op*. *cit*., p. 10.

6 Ranmal A. Samarasinghe *et al*., *Identification of Neural Oscillations and Epileptiform Changes in Human Brain Organoids*, 24 Nat. Neurosci. 1488–1500 (2021).

7 Lavazza & Pizzetti *op*. *cit*., pp. 10–1; Taupitz *op*. *cit*., pp. 101–2.

8 This also means that human brain organoids cannot have the special legal status that is given to embryos—in the European Union, for instance, embryos are classified as ‘legal subjects.’ Currently, the legal status of human brain organoids is generally the same as that of other human biological materials.

9 Alysson Renato Muotri, *Applications of Human Brain Organoids*, 2 Organoid e13 (2022).

10 Elke Gabriel *et al*., *Human Brain Organoids Assemble Functionally Integrated Bilateral Optic Vesicles*, 28 Cell Stem Cell 1740–57 (2021).

11 Jimena Andersen *et al*., *Generation of Functional Human 3D Cortico-Motor Assembloids*, 183 Cell 1913–29 (2020).

12 Aleksander Skardal *et al*., *Drug Compound Screening in Single and Integrated Multi-Organoid Body-On-A-Chip Systems*, 12 Biofabrication 025017 (2020).

13 This law has been criticized from the ‘pro-choice’ position because it applies to failed abortion. This is an instance of our claim that laws related to abortion have important implications for the issue of the natural personhood of brain organoids, as discussed below.

14
Sensenbrenner Jr. & Hon F. James, Report From the Committee on the Judiciary: Born-Alive Infants Protection Act of 2001 (2001), https://www.congress.gov/congressional-report/107th-congress/house-report/186 (accessed March 6, 2023).

15 Nobuyuki Eura *et al*., *Brainstem Organoids from Human Pluripotent Stem Cells*, 14 Front. Neurosci. 538 (2020).

16 This can be seen as an example of a more general normative problem: whether there should be differences in the legal treatment of similar end products from different processes (thanks to reviewers for this point). For example, in the United States, until recently meat was defined as flesh taken from a carcass, leading to regulatory problems of lab-grown meat (thanks to a reviewer for providing this example). As another example, blastocyst-like structures have recently been produced from pluripotent stem cells, and there is international disagreement as to whether such ‘blastoids’ should be regulated in the same way as blastocysts. See, Tsutomu Sawai, Kyoko Akatsuka, Go Okui & Tomohiro Minakawa, *The Regulation of Human Blastoid Research: A Bioethical Discussion of the Limits of Regulation*, 23 Embo Rep. e56045 (2022). Fetal surgery and artificial wombs, which we discuss briefly below, would be technologies raising the same issue. Our basic assumption is that differences in process should not affect the legal treatment of the product. For this reason, we do not consider the possibility that the process of being grown in a laboratory *per se* affects the legal status of human brain organoids. In fact, to the best of our knowledge, no such claim has ever been made. However, this is a possible theoretical position, which should be further explored in light of the other similar cases mentioned above.

17 Elizabeth Chloe Romanis, *Challenging the “Born Alive” Threshold: Fetal Surgery, Artificial Wombs, and the English Approach to Legal Personhood*, 28 Med. Law Rev. 93–123 (2020).

18 Emily A. Partridge *et al*., *An Extra-Uterine System to Physiologically Support the Extreme Premature Lamb*, 8 Nat. Commun. 15112 (2017).

19 Lavazza & Pizzetti *op*. *cit*.; Hostiuc, Rusu, Negoi, Perlea, Dorobanţu & Drima *op*. *cit*.; Julian Koplin, Olivia Carter & Julian Savulescu, *Moral Status of Brain Organoids*, in Rethinking Moral Status 205–68 (Steve Clarke, Hazem Zohny & Juliam Savulescu eds, 2021).

20 We borrow this argumentative strategy from Simon Chesterman, *Artificial Intelligence and the Limits of Legal Personality*, 69 Int. Comp. Law Q. 819–44 (2020). Chesterman examines whether the following three theories can be applied to artificial intelligence systems.

21 The following characterizations of the three theories of corporate personhood are based on Susanna Kim Ripken, Corporate Personhood (2019).

22 Tsutomu Sawai, Hideya Sakaguchi, Elizabeth Thomas, Jun Takahashi & Misao Fujita, *The Ethics of Cerebral Organoid Research: Being Conscious of Consciousness*, 13 Stem Cell Rep. 440–7 (2019).

23 Lavazza & Pizzetti *op*. *cit*.

24 John Seewer, *US judge: Pablo Escobar’s cocaine hippos legally “people”*, Ap News, October 26, 2021, https://apnews.com/article/hippos-animals-personhood-pablo-escobar-e89daf05efb37efd3d35e6dabce56726. (accessed November 8, 2022).

25 Lavazza & Pizzetti *op*. *cit*. If sentient human brain organoids are created, it is unclear whether the public will have the same emotional response to them as to animals. Speculatively, however, their human origin may be very disturbing to the public. Still, because they lack the obvious pain behaviors, unlike animals, the public may not empathize with them as much. To date, there has been little empirical research on public attitudes toward human brain organoids. Such research will be important for further legal consideration of human brain organoid research.

26 For examples of various rivers given juridical personhood and a comparative analysis of them, see Cristy Clark, Nia Emmanouil, John Page & Alessandro Pelizzon, *Can You Hear the Rivers Sing? Legal Personhood, Ontology, and the Nitty-Gritty of Governance*, 45 Ecol. Law Q. 787–844 (2018).

27 Abigail Hutchison, *The Whanganui River as a Legal Person*, 39 Altern. Law J. 179–82 (2014).

28 Juli Bollinger, Elizabeth May, Debra Mathews, Mark Donowitz & Jeremy Sugarman, *Patients’ Perspectives on the Derivation and Use of Organoids*, 16 Stem Cell Rep. 1874–83 (2021); Haselager, Boers, Jongsma, Vinkers, Broekman & Bredenoord *op*. *cit*.

29 Some may be concerned that such a legal decision will reflect social beliefs but not the scientific facts about human brain organoids. However, what the river cases demonstrate is precisely that the recognition of juridical personhood is not always based solely on ‘scientific’ facts about the object. As to the *normative* question of whether it is appropriate to grant juridical personhood in this way, we refrain from making a judgment here.

30 In addition, as noted with respect to animals, public attitudes toward human brain organoids could also provide a rationale for their legal protection, leading to recognition of their juridical personhood.

31 Brett J. Kagan *et al*., *In Vitro Neurons Learn and Exhibit Sentience When Embodied in a Simulated Game-World*, 110 Neuron 1–18 (2022).

32 Julian Savulescu, Christopher Gyngell & Tsutomu Sawai, *Tech firms are making computer chips with human cells: is it ethical?*, Conversation, May 24, 2022, https://theconversation.com/tech-firms-are-making-computer-chips-with-human-cells-is-it-ethical-183394. (accessed November 8, 2022).

33 Strictly speaking, what is at issue here is not the juridical personhood of brain organoids themselves but that of the hybrid system composed of brain organoids and machines. However, as we will see just below, comparisons with AI systems are often made in discussions about the legal personhood of human brain organoids, so that we will touch upon this topic in this paper. Note that for the same reason of focusing on the brain organoids themselves, this paper has not addressed the legal personhood of animals with transplanted human brain organoids. If, as is often concerned, the transplantation results in cognitive enhancement, it would also enhance the interests of the host animals. To protect them, juridical personhood could be granted to such chimeras. Some also believe that the existence of chimeras would invalidate the very idea of legal personhood, and human brain organoid transplants may provide further support for such a view. See, Tomasz Pietrzykowski, Personhood Beyond Humanism: Animals, Chimeras, Autonomous Agents and the Law (2019).

34 European Parliament, Resolution of 16 February 2017 with recommendations to the Commission on civil law rules on robotics [2015/2103(INL)]. (2017), https://www.europarl.europa.eu/doceo/document/TA-8-2017-0051_EN.html (accessed March 6, 2023). In a more recent report, however, the European Parliament appears to have become more cautious about granting juridical personhood to AI systems. This illustrates how fluid this issue is now. For a recent view, see European Parliament, Report with recommendations to the Commission on a civil liability regime for artificial intelligence [2020/20140(INL)] (2020), https://www.europarl.europa.eu/doceo/document/A-9-2020-0178_EN.html (accessed March 6, 2023).

35 Chesterman *op*. *cit*.

36 Lavazza & Pizzetti *op*. *cit*. n. 43.

37 Acknowledging this does not affect our argument in support of the possible natural personhood of human brain organoids in Section II.B. There, we argued that human brain organoids are *not* mere body parts, but rather have the potential to be classified as human beings as a whole.

38 We would like to add that even if human brain organoids are legal ‘properties,’ rather than legal persons, this does not mean that anything goes in the accompanying research. As many researchers have pointed out, in the event that human brain organoids were to be classified as legal properties, if the organoids were able to feel pain or pleasure, their welfare would need to be protected, as is the case with laboratory animals. See, for example, Julian J. Koplin & Julian Savulescu, *Moral Limits of Brain Organoid Research*, 47 J. Law Med. Ethics 760–7 (2019).

39 For such legal issues, see the contributions in Dederer and Hamburger (eds.) *op*. *cit*.

